# A spatial equity analysis of a public health intervention: a case study of an outdoor walking group provider within local authorities in England

**DOI:** 10.1186/s12939-015-0256-x

**Published:** 2015-10-29

**Authors:** Sarah Hanson, Andy Jones

**Affiliations:** Norwich Medical School, University of East Anglia, Norwich, Norfolk NR4 7TJ UK

**Keywords:** Physical activity, Walking groups, Health inequity, Spatial equity, Public health

## Abstract

**Introduction:**

If an intervention is not well spatially targeted, appropriate levels of uptake, efficacy, long-term compliance and improved health outcomes are unlikely to be attained. Effective health interventions should seek to achieve not only absolute improvements in health but also to reduce inequity. There is often a disparity whereby preventative interventions are more likely to be successful amongst the more affluent, a process which has been coined the ‘inverse prevention law’. Physical inactivity is known to be socially patterned and disproportionately prevalent in disadvantaged communities yet there is a lack of clear evidence on which interventions have the potential to influence inequity.

Walking groups have been found to have multiple health benefits and increase physical activity. In England the major facilitator is a not for profit organisation which has 70,000 regular walkers and is lay led with 10,000 volunteers. The aim of this study was to evaluate the extent to which walking groups operated in those places with the greatest health need and whether consequently the scheme has the potential to influence health inequity.

**Method:**

The work used a spatial approach whereby geographical variations in walking group provision within the 326 local authorities in England (mean population 163,410) were linked to health and socio-economic measures of population need.

**Results:**

Generally, greater need was not associated with higher provision of the walking group intervention. Although the magnitude of differences was small, provision of the intervention tended to be poorest in those local authorities with the greatest health need, as measured by our indicators.

**Conclusions:**

Without targeting those areas with greater health and socio-economic need, there is a concern that walking groups may not be set up in areas that need them most. There is therefore a potential that this intervention could, albeit in a small way, widen inequity between local authorities. However small-scale and well-intentioned, interventions need to be evaluated for their potential impact on inequity.

## Introduction

The term health inequity describes the unnecessary and avoidable inequalities in health between different groups of people. These inequities arise from inequalities within and between societies and are considered unfair and unjust [1, 2]. There are important social and economic costs to society from health inequity, as well as it being a matter of social justice [1, 3, 4].

Inequity in health is a problem in all developed countries [5]. In the United Kingdom (UK), the ‘Black Report’ of 1980 was the first to detail the extent of which ill-health and death were unequally distributed [6]. These inequalities were not due solely to failings in the National Health Service (NHS) but to many other social determinants of health. Furthermore, there was evidence that these inequalities were widening [7]. This trend continues to the present day with an estimate of between 1.3 and 2.5 million extra years of life lost each year in England due to health inequalities [3].

Determinants of poor health extend beyond individual characteristics into environmental settings, such as workplaces and neighbourhoods (residential settings), so that where you live matters [8]. Importantly, differences across neighbourhoods are not in themselves natural; rather they result from specific policies, or the absence of policies [9]. Therefore, targeting spatial inequalities within neighbourhoods is an attractive target for interventions [10]. However, there is a concern that many interventions designed to improve health may not be reaching the most disadvantaged, as was recognised by the UK Government following the publication of the ‘Wanless Report’ of 2004 [11]. It has been observed that there is often a disparity whereby uptake and provision of preventative interventions is socially patterned and are more likely to be successful amongst the more affluent, a process which has been coined the ‘inverse prevention law’ [12]. Indeed some researchers have pointed to evidence of an ‘inverse equity hypothesis’ whereby, in the absence of effort to promote equity, new health interventions will first be adopted by the wealthy, but as coverage increases poorer individuals will make faster gains and initial inequity would eventually be reduced [13]. Others have shown evidence of cumulative inequity, expressed as ‘the staircase effect’, whereby cumulative disadvantage at different stages of interventions acts to decrease the effectiveness of any potential benefits [14]. There is also some evidence that those interventions that rely on voluntary behaviour change, as opposed to statutory regulation, can have a particularly negative impact on health equity because they will tend to be adopted by those with the least need [15, 16].

Many population health interventions focus on unhealthy behaviours which are a key contributor to non-communicable disease mortality and disease burden. Such diseases are largely preventable but are disproportionately prevalent in poor and disadvantaged communities, with evidence that this disparity is increasing [17, 18].

Physical activity is known to have wide ranging long term health benefits across all socio-economic and ethnic groups and sexes [19, 20]. A key component to unhealthy behaviour is physical inactivity, which has been estimated to be associated with 9 % of premature deaths worldwide [21]. Physical inactivity has been estimated to cost the English National Health Service (NHS) £1.06 billion per year in direct costs, with lost productivity an estimated £6.5 billion per year [22]. For example, it is one of the major risk factors for cardiovascular disease; one of the major causes of premature death in England and associated with 34 % of all deaths [23]. It is of concern that it has been shown that a higher prevalence of leisure-time or moderate–vigorous intensity physical activity is found in the most educated [24]. Further, economically disadvantaged and vulnerable groups are less likely to engage with physical activity interventions [25]. Accessibility of physical activity interventions may be one of several environmental factors that influence individuals’ physical activity behaviours [26]. An evaluation of physical activity interventions in Europe found that very few of the analysed policies included specific measures to increase participation of economically disadvantaged population groups [27]. There is therefore a risk that interventions to increase physical activity could increase health inequity, representing a major challenge to public health professionals.

Outdoor walking group schemes are a widely recommended as a way of increasing physical activity and have been shown to be effective at the population level [28–30]. A recent systematic review found them to have numerous health benefits with virtually no adverse effects [31]. They are also efficacious at increasing physical activity, particularly when targeted at older adults [32]. Furthermore, group based physical activity interventions aimed at adults have previously been found to be effective in socio-economically disadvantaged communities [33].

In England the major facilitator of group walking schemes is the national ‘Walking for Health’ (WfH) programme [34]. The concept of group health walks in England was introduced by an Oxfordshire General Practitioner in 1995, developing into a national ‘Walking for Health’ scheme in 2000. It is run by two charities. WfH is England’s largest network of lay-led health group walks with 70,000 regular walkers, 10,000 volunteer walk leaders and approximately 3,000 free short walks weekly in the natural environment. The stated aims are to ‘provide a local, low cost, fun, social method of becoming active’ with those at highest risk of inactivity being particularly targeted [34].

The scheme is organised centrally with salaried co-ordinators but it is primarily a community delivered intervention, funded locally through partnership arrangements. The group walks are delivered by volunteers. Volunteers receive one day of standardised training and then lead walks in their own community. Information on how to set up or add to existing schemes is given on the scheme’s website and it is open to members of the public to apply to do this. There are requirements for accreditation and for using the scheme branding. This includes that all walk leaders receive training and that the walks are free and regular and should be no longer than 90 min in length, with at least one 30 min walk per month. Information is also given on the scheme website about how to apply for local funding from sources such as local authorities and NHS commissioners. The scheme therefore presents a case-study opportunity to evaluate the extent to which such an intervention that is nationally organised but delivered locally on a voluntary basis operates in those places with the greatest health need and has the potential to influence inequity.

This work takes a spatial approach, whereby geographical variations in walking group provision in England are linked and then compared to variations in a range of measures of population need. Taking such an approach is appropriate as it has been argued that the achievement of spatial equity is the first step in a process towards reducing health inequity; if an intervention is not well spatially targeted, appropriate levels of uptake, efficacy, long-term compliance, and the accomplishment of improved health outcomes are unlikely to be attained [35].

## Methods

### Walking for Health (WfH) scheme data

To evaluate the extent to which WfH operates in those places with the greatest need it was firstly necessary to identify where WfH walks were provided. England is organised administratively into 326 local authorities (LAs) with a mean population of 163,410 [36]. This is the administrative area that the WfH scheme uses to collate its data and therefore local authorities were used as the spatial units for this analysis. For the purposes of this research the intervention is defined as the operation of a group walk. Each local WfH scheme records these walks onto a national database. The measure of provision therefore was the number of group walks recorded on the national database within each of the 326 local authorities in England over a 12 month period. Data was extracted in September 2014 for walks registered between the 1^st^ April 2013 to 31^st^ March 2014, the period for which most recent complete information was available.

### Health and socio-economic measures in Local Authorities (LAs)

A set of variables were generated to describe the level of health and socio-economic need for the provision of physical activity interventions within each local authority. Two sets of indicators were generated. Firstly, data was chosen to represent direct health need. This included behavioural measures such as physical inactivity. Secondly, as it is widely recognised that some socio-demographic groups can be disadvantaged in health programmes, demographic indicators were chosen that represent these socio-economic factors. The Public Health Observatory Handbook of Health Inequalities was used to provide guidance on the selection of appropriate measures [37].

The full list of variables representing need are listed in Table 1. All data was derived from routinely available national datasets. The data used to generate the variables was obtained from the Office for National Statistics which provides information on the age and socio-economic makeup of the population via the national census of population as well as general health [38], from Public Health England and the Public Health Observatories for health and health care data [39] and from the Active People Survey [40] for recreational physical activity data.

**Table 1:** Variables generated to describe health and socio-economic measures within each local authority

	Description of the variable and the unit it is expressed in
1. Measure of health need	
Aged above 65^a^.	Persons aged above 65 years of age as a percentage of the total population.
Physically inactive < 30 min per week^b^.	Percentage of all adults (aged 16 and over) participating in sport and/or undertaking some form of physical activity at moderate intensity (or higher) in 10 min blocks. Includes recreational walking and walking for active travel.
Mortality^c^.	All cause standardised mortality ratios (SMR). SMR is based on an England standard of 100. Greater than 100 is greater than national average.
Inequality in life expectancy (For male and females)^d^.	Slope index of inequality (SII) for life expectancy at birth using Index of Multiple Deprivation data (2010) and mortality data for 2006–10. Hypothetical difference in life expectancy within a district expressed in years.
Limiting long term illness or disability which limits daily activity or work^a^.	Percentage of people with day to day activities limited. Self-rated. A percentage of the total number of respondents to the question.
Self-rated health^a^.	Self-rated health. The percentage of the total population rating their health bad and very bad.
Chronic and poorly managed diseases: Chronic obstructive pulmonary disease (COPD) and Coronary heart disease (CHD)^e^.	Emergency admissions for COPD and Emergency admissions for CHD. Standardized admission ratio 100 is the England benchmark.
Excess weight (Body Mass Index (BMI) ≥ 25 kg/m^2^)^b^.	Self-reported height and weight information (BMI) . Adults, 16 years and over. Uses Health Survey for England methodology for adjusting for inaccuracy in self-reporting. A percentage estimate of the prevalence of excess weight.
2. Socio-economic measures	
Socio-economically disadvantaged adults^f^.	English Index of Multiple Deprivation (IMD) 2010. Population weighted average of the combined scores for the lower super output areas in a local authority. Lower is less disadvantage.
Income deprivation amongst adults^g^.	Income domain from IMD 2010. Percentage of the total population living in low income families (out of work and low income dependent on means dependent benefits).
Socio-economic disadvantage in older people^h^.	Income Deprivation Affecting Older People (IDAOP) Percentage of adults aged 60 or over living in pension credit households as a percentage of all adults aged 60 or over.
Pensioners living alone^a^.	People aged 65 or over living alone as a percentage of all adults aged 65 or over.
Black and Minority Ethnic (BME) adults^a^.	Non-white as a percentage of the total adult population.

^a^Census 2011 Available at: http://www.ons.gov.uk/ons/guide-method/census/2011/index.html

^b^Active People Survey (data collected between January 2012 and January 2013) commissioned by Sport England Available at: http://archive.sportengland.org/research/active_people_survey.aspx

^c^Office for National statistics 2012 Available at: http://www.ons.gov.uk/ons/publications/re-reference-tables.html?edition=tcm%3A77-314473

^d^Public Health England 2013 Available at: http://www.apho.org.uk/default.aspx?RID=110504

^e^Hospital episode statistics from Public Health England. Admissions from April 2006 to March 2011 Available at: http://www.yhpho.org.uk/default.aspx?RID=8494

^f^Social Disadvantage Research Centre at the Department of Social Policy and Social work at the University of Oxford. Commissioned by the Department for Communities and local government March 2011 Available at: https://www.gov.uk/government/statistics/english-indices-of-deprivation-2010

^g^Public Health England Commissioned by the Department for Communities and local government Available at: http://www.apho.org.uk/resource/item.aspx?RID=97316

^h^Social Disadvantage Research Centre at the Department of Social Policy and Social work at the University of Oxford. Commissioned by the Department for Communities and local government March 2011 Available at: http://www.apho.org.uk/resource/item.aspx?RID=97318

### Statistical analysis

Initial data extraction showed there was no evidence of group walk provision in 128 of the 326 LAs; a large percentage (39.2 %). Therefore we firstly wanted to determine any differences in the health and socio-economic measures between those LAs with the provision and those without it. For each of these measures, the differences in mean values were compared in those LAs with walks and those without. Analysis of variance was used to test the difference in means with an odds ratio computed using binary logistic regression.

The second component of this analysis examined those LAs where there was evidence of the group walks. The aim of this was to determine whether the number of walks recorded in these LAs was associated with the health and socio-economic measures. In order to do this, the mean number of group walks over the study period was classified into quintiles representing least to most group walks. Trends in the mean values of the variables across quintiles were examined using a test for linear trend by means of polynomial contrast. The threshold for statistical significance was *p* = 0.05. SPSS Version 22 was used for all analysis [41].

## Results

Based on the information extracted from the national database, between April 2013 and March 2014 the WfH scheme provided 58,525 walks in England with 48,277 registered walkers and a total attendance at walks of 856,239 people. The population of walkers was of an older age group; 81 % were aged above 55 years of age and 48 % above 65. There was evidence of the WfH intervention operating in 198 of the 326 (61 %) of the local authorities. Where the group walks operated, the median was 225 group walks per year. This ranged from < 10 (6 LAs) to 2037 (1 LA) group walks in the year. The interquartile range was from 83 to 408 group walks.

Table 2 describes the difference in the health and socio-economic measures between those local authorities with no evidence of the WfH intervention and those with it. In general, those local authorities with no evidence of WfH provision were more likely to have greater need, as measured by the health and socio-economic indicators, with statistically significant differences for 10 of the 15 measures. There was WfH provision in those LAs with districts with greater populations aged above 65 and with limiting long term illness. Otherwise there was a greater odds of no provision in those LAs with greater socio-economic and health need as represented by our health and socio-economic measures. The size for these differences were however modest with odds ratios relatively close to unity.

**Table 2:** Difference in health and socio-economic measures between local authorities (LA) with and without the intervention

	Unit	LA No provision	LA provision	*p*-value*	Odds ratio^a^ (95 % CI)
		*n* = 128	*n* = 198		
		Mean value (95 % CI)	Mean value (95 % CI)		
1. Measure of health need					
Above age 65	%	16.22 (15.52 to 16.92)	18.22 (17.67 to 18.77)	<0.001	1.14 (1.07 to 1.2)
Physically inactive	%	28.06 (27.19 to 28.93)	27.69 (27.14 to 28.23)	0.45	0.980 (0.93 to 1.03)
Standardised mortality ratio	Ratio	98.67 (96.14 to 101.2)	96.78 (95.05 to 98.97)	0.21	0.99 (0.97 to 1.01)
Inequality in life expectancy SII (males)	Years	8.06 (7.54 to 8.58)	7.34 (6.98 to 7.97)	0.04	0.924 (0.86 to 0.99)
Inequality in life expectancy SII (females)	Years	5.59 (5.15 to 6.03)	5.35 (5.0 to 5.7)	0.40	0.962 (0.88 to 1.05)
Limiting long term illness or disability	%	17.08 (16.51 to 17.65)	18.03 (17.6 to 18.5)	0.01	1.101 (1.02 to 1.18)
Bad and very bad health	%	5.25 (5.00 to 5.50)	5.28 (5.09 to 5.46)	0.87	1.013 (0.86 to 1.19)
Chronic and poorly managed disease COPD	Ratio	102.16 (94.33 to 109.98)	88.03 (82.91 to 93.16)	0.002	0.991 (0.986 to 0.997)
Chronic and poorly managed disease CHD	Ratio	100.40 (96.10 to 104.72)	95.12 (92.17 to 98.07)	0.04	0.990 (0.98 to 1.00)
Excess weight (BMI ≥ 25 kg/m^2^)	%	64.27 (63.31 to 65.23)	64.30 (63.59 to 65.02)	0.95	1.001 (0.96 to 1.04)
2. Socio-economic measures					
Index of multiple deprivation	Average score	20.59 (18.91 to 22.26)	18.23 (17.18 to 19.27)	0.01	0.967 (0.94 to 0.99)
Income domain IMD	%	13.86 (12.75 to 14.97)	12.45 (11.78 to 13.11)	0.02	0.954 (0.92 to 0.99)
Income deprivation older people	%	18.40 (16.98 to 19.83)	16.21 (15.41 to 17.02)	0.01	0.954 (0.92 to 0.99)
Pensioners living alone	%	31.97 (31.30 to 32.64)	30.77 (30.30 to 31.24)	0.003	0.910 (0.85 to 0.97)
Non-white	%	14.78 (12.2 to 17.37)	8.02 (6.52 to 9.53)	<0.001	0.958 (0.94 to 0.98)

*Abbreviations*: *SII* Slope index of inequality, *COPD* Chronic obstructive pulmonary disease, *CHD* Coronary heart disease, *IMD* Index of multiple deprivation

*Based on analysis of variance to test the difference in means

^a^An odds ratio generated using binary logistic regression

Table 3 shows trends in the level of walk provision across the health and socio-economic measures for those LAs within which at least some walk provision was present. Generally, poorer health and socio-economic measures in LAs was not associated with a trend of higher walk provision, except for the measure of the percentage of older people resident in the LA. There was some evidence that provision was greater in areas with more limiting long term illness or disability and poorer self-rated health but trends did not reach statistical significance. For all other indicators, provision was generally poorer in areas of greater need, although trends only reached statistical significance for the measures of pensioners living alone and emergency admissions for COPD, and the magnitude of differences in mean values between the LAs with the most and least number of walks were again small.

**Table 3:** Health and socio-economic measures for each quintile of intervention in local authorities with the intervention (*n* = local authority)

		Group 1	Group 2	Group 3	Group 4	Group 5	Test for linear trend^a^
		LAs with least provision				LAs with most provision	
		Mean value (95 % CI)	Mean value (95 % CI)	Mean value (95 % CI)	Mean value (95 % CI)	Mean value (95 % CI)	*p*-value
		*n* = 39	*n* = 40	*n* = 40	*n* = 40	*n* = 39	
1. Measure of health need							
Above aged 65	%	16.97 (15.43 to 18.52)	17.94 (16.72 to 19.17)	18.93 (17.74 to 20.12)	18.61 (17.52 to 19.70)	18.62 (17.42 to 19.83)	0.046
Physically inactive (<30 min per week)	%	28.37 (27.07 to 29.67)	27.52 (26.28 to 28.76)	26.99 (25.86 to 28.13)	27.33 (26.19 to 28.47)	28.25 (26.86 to 29.64)	0.826
Standardised mortality ratio	Ratio	98.69 (95.11 to 102.27)	95.25 (89.36 to 101.14)	96.33 (92.75 to 99.90)	95.38 (91.93 to 98.82)	98.36 (95.75 to 100.96)	0.931
Inequality in life expectancy SII (males)	Years	7.81 (6.85 to 8.77)	7.39 (6.46 to 8.31)	6.62 (5.73 to 7.52)	8.01 (7.00 to 9.02)	7.08 (6.24 to 7.93)	0.574
Inequality in life expectancy SII (females)	Years	5.94 (5.03 to 6.84)	5.59 (4.83 to 6.35)	4.73 (3.88 to 5.57)	5.63 (4.74 to 6.52)	4.87 (4.29 to 5.44)	0.098
Limiting long term illness or disability	%	17.64 (16.58 to 18.69)	17.99 (17.02 to 18.98)	17.94 (16.91 to 18.98)	18.23 (17.29 to 19.16)	18.34 (17.32 to 19.36)	0.299
Self-rated bad health and very bad health	%	5.28 (4.88 to 5.69)	5.33 (4.89 to 5.76)	5.14 (4.64 to 5.63)	5.31 (4.90 to 5.72)	5.33 (4.92 to 5.74)	0.912
Chronic and poorly managed disease COPD	Ratio	100.91 (88.64 to 113.19)	89.73 (78.66 to 100.81)	81.11 (68.07 to 94.15)	87.04 (75.45 to 98.64)	81.52 (71.58 to 91.45)	0.024
Chronic and poorly managed disease CHD	Ratio	98.02 (91.50 to 104.53)	96.19 (89.78 to 102.60)	91.41 (84.07 to 98.74)	95.80 (90.04 to 101.56)	94.24 (86.44 to 102.04)	0.457
Excess weight (BMI ≥ 25 kg/m^2^)	%	63.71 (62.21 to 65.21)	65.58 (63.79 to 67.37)	64.68 (63.27 to 66.10)	64.79 (63.13 to 66.46)	62.69 (60.93 to 64.46)	0.272
2. Socio-economic measures							
Index of multiple deprivation	Average score	19.63 (17.09 to 22.16)	18.09 (15.82 to 20.37)	16.49 (13.95 to 19.04)	18.22 (16.09 to20.42)	18.74 (16.34 to 21.15)	0.663
Income domain IMD	%	12.99 (11.24 to 14.74)	12.44 (10.93 to 13.96)	11.37 (9.87 to 12.86)	12.56 (11.15 to 13.96)	12.90 (11.45 to 14.35)	0.980
Income deprivation older people	%	17.60 (15.32 to 19.88)	16.32 (14.32 to 18.33)	14.94 (13.19 to 16.69)	15.85 (14.38 to 17.31)	16.40 (14.79 to 18.00)	0.321
Pensioners living alone	%	31.25 (30.33 to 32.17)	31.78 (30.33 to 33.23)	30.06 (29.18 to 30.93)	30.66 (29.70 to 31.63)	30.11 (29.09 to 31.12	0.044
Non-white	%	11.26 (6.35 to 16.18)	7.84 (5.49 to 10.20)	7.19 (3.74 to 10.65)	7.13 (4.00 to 10.25)	6.75 (3.89 to 9.61)	0.073

*Abbreviations*: *SII* Slope index of inequality, *COPD* Chronic obstructive pulmonary disease, *CHD* Coronary heart disease, *IMD* Index of multiple deprivation

^a^Polynomial contrast for linear trend across quintiles

## Discussion

The magnitude of differences in this case study were small, but the findings showed that the number of the group walks provided was generally lowest in those local authorities with the greatest health need, as measured by our health and socio-economic indicators. The only statistically significant exception to this observation was that provision of walks was better in those LAs areas with greater numbers of older people, although not for pensioners living alone. Health walks are predominantly attended by older people and therefore the scheme could be well positioned to have a positive impact on the physical activity levels of older people, a group at particular risk of inactivity [42]. Group walks may help address the social isolation, loneliness and higher levels of deprivation that are linked with pensioners who live alone [43]. Indeed, there is evidence of an income-age gradient in physical activity with the largest differences by income occurring in those who are up to 10 years post statutory retirement age [44]. It is of concern however, that walk provision was generally poorer in areas with the greatest need measured by the other health and socio-economic indicators.

It is probably the case that the measures we used to describe each LA captured an overall health and socio-economic disadvantage in certain areas and that some of the measures we used were better at picking this up than others. As a consequence specific associations with individual measures need to be interpreted with caution. However, it is noteworthy that where there was no evidence of the walk provision being provided at all, this tended to be in those LAs with a poorer health and socio-economic profile across a wide range of measures. This is despite the fact that the organisation in this case study encourages the start-up of new schemes, and it offers information to assist those who might want to set up new group walks in their community on how to apply for local funding from sources such as local public health and the NHS. It is thus of concern that the initial impetus to instigate the scheme appears is most lacking in deprived areas rather than limitations in the provision of new walks in localities where some already present. For the scheme to operate more universally the particular barriers involved in starting walks when none are present may need to be addressed.

The intervention considered in this case study started as a small local initiative which has grown organically into a large national organisation operating in both urban and rural communities. WfH recognises that physical inactivity is an increasing challenge and that more health walks are needed to reach as many people as possible. However, previous research has cautioned that walking interventions may be preferentially taken up by better-off groups [45]. For example, research with one walking group organisation found that 72 % of the membership were professionals with new members attracted by ‘word of mouth’. This subsequently attracted people from similar demographics—the retired, middle class and largely female [46]. Community participation is key to health promotion and to reach into those that are more disadvantaged there is a need to better mobilise the energy and resources that comes from within communities [47]. Previous research has identified involving residents in a bottom up approach with meaningful engagement and the support of volunteers as key to successful physical activity interventions [48]. Additionally, the use of lay community/lay volunteers has shown some promise in improving health amongst disadvantaged groups in general [49] with specific successes in mental health and lifestyle improvement [50]. It might be that a model of partnership working with a community health champion approach could aid productive access into disadvantaged areas.

## Strengths and limitations

A strength of this analysis is the wide variety of datasets used to generate health and socio-economic measures within local authorities. However, as a consequence we undertook a large number of tests which raises the potential of type I statistical error associated with multiple testing. The study also benefitted from access to a large national database of standardised measures of walk provision. In common with any analysis that makes use of an organisation’s database, we are vulnerable to incompleteness. Our discussion with the WfH scheme suggests there may be some missingness in the data where some schemes use their own database software which is not compatible with the central database we had access to. There is however no suggestion that this missingness is more prevalent in more disadvantaged areas. A further limitation is that our health and socio-economic measures were area based and hence did not provide any insight into the health needs of those individuals who actually attend the walks. LAs in England have a mean population of 163,410 [36]. Both rural and metropolitan LAs are very heterogeneous and therefore our findings for a LA cannot be taken as a proxy for more local neighbourhoods [9]. Finally, the findings from this analysis are limited to one health intervention in one country. Caution should therefore be given to how generalizable these findings are to other settings.

## Conclusions and implications of this study

Our study showed some inequity between LAs in walking group provision. There was no evidence of higher levels of provision in areas of greatest need and they also tended to operate in those areas that have better socio-economic and health indicators. The magnitude of the differences were small and on their own unlikely to meaningfully contribute to health inequity. However, if these findings were similarly replicated in other health initiatives they could act additively and lead to significant inequity in final outcomes (White et al., [15]). It has previously been cautioned that all processes in the planning and delivery of an intervention have the potential to widen inequity between groups [15]. Our study has shown that it is possible that the way walking schemes are developed through local initiatives could create the potential to widen inequity between local authorities as they might not be set up in those communities that stand to gain the most.

## References

[1] Whitehead M (1992). The concepts and principles of equity and health. A fair chance for all.

[2] Social determinants of health. [http://www.who.int/social_determinants/thecommission/finalreport/key_concepts/en/]. Accessed 29 July 2015.

[3] Marmot M, Allen J, Goldblatt P, Boyce T, McNeish D, Grady M, Geddes I (2010). Fair society, healthy lives: Strategic review of health inequalities in England post-2010.

[4] Wilkinson R, Pickett K. The spirit level: Why equality is better for everyone. London: Penguin UK; 2010

[5] Closing the Health Inequalities Gap: An International Perspective. [http://www.euro.who.int/__data/assets/pdf_file/0005/124529/E87934.pdf]. Accessed 31 October 2014.

[6] Department of Health and Social Security (1980). Inequalities in Health: Report of a Working Group Chaired by Sir Douglas Black.

[7] Townsend P, Davidson N, Whitehead M. Inequalities in health: the Black report. London: Penguin Books Great Britain; 1992.

[8] Cummins S. Chapter 18. Improving population health through area-based social interventions: generating evidence in a complex world. In Evidence-based public health: effectiveness and efficiency. Edited by Killoran A. Oxford: Oxford University Press; 2010.

[9] Diez-Roux A-V (2007). Neighborhoods and health: where are we and were do we go from here?. Rev Epidemiol Sante Publique.

[10] Diez Roux AV, Mair C (2010). Neighborhoods and health. Ann N Y Acad Sci.

[11] Treasury HMs (2004). The Wanless report: Securing good health for the whole population. HM Treasury, London.

[12] Acheson D. Independent Inquiry into Inequalities in Health Report. London: Department of Health; 1998.

[13] Victora CG, Vaughan JP, Barros FC, Silva AC, Tomasi E (2000). Explaining trends in inequities: evidence from Brazilian child health studies. The Lancet.

[14] Tugwell P, de Savigny D, Hawker G, Robinson V (2006). Applying clinical epidemiological methods to health equity: the equity effectiveness loop. BMJ.

[15] White M, Adams J, Heywood P. How and why do interventions that increase health overall widen inequalities within populations? Social Inequality and Public Health. Edited by Savatore JB. Chapter 5. The Policy Press in Bristol; 2009:65–82

[16] Lorenc T, Petticrew M, Welch V, Tugwell P (2013). What types of interventions generate inequalities? Evidence from systematic reviews. J Epidemiol Community Health.

[17] Buck D, Frosini F (2012). Clustering of unhealthy behaviours over time: Implications for policy and practice.

[18] WHO. 2008–2013 Action Plan for the Global Strategy for the Prevention and Control of Noncommunicable Diseases. Geneva; 2008.

[19] Hallal PC, Bauman AE, Heath GW, Lee I-M, Pratt M (2012). Physical activity: more of the same is not enough. The Lancet.

[20] Reiner M, Niermann C, Jekauc D, Woll A (2013). Long-term health benefits of physical activity--a systematic review of longitudinal studies. BMC Public Health.

[21] Lee IM, Shiroma EJ, Lobelo F, Puska P, Blair SN, Katzmarzyk PT (2012). Effect of physical inactivity on major non-communicable diseases worldwide: an analysis of burden of disease and life expectancy. The Lancet.

[22] Physical activity (NICE guidance LGB3). [http://www.nice.org.uk/advice/lgb3/chapter/Costs-and-savings]. Accessed 13 July 2015.

[23] Prevention of cardiovascular disease (NICE guideline PH25). [http://www.nice.org.uk/guidance/ph25/chapter/2-Public-health-need-and-practice]. Accessed 13 July 2015.

[24] Gidlow C, Johnston LH, Crone D, Ellis N, James D (2006). A systematic review of the relationship between socio-economic position and physical activity. Health Educ J.

[25] Hillsdon M, Lawlor D, Ebrahim S, Morris J (2008). Physical activity in older women: associations with area deprivation and with socioeconomic position over the life course: observations in the British Women’s Heart and Health Study. J Epidemiol Community Health.

[26] Diez-Roux AV, Moore L, Evenson KR, McGinn AP, Brown DG, Brines S, Jacobs DR (2007). Availability of Recreational Resources and Physical Activity in Adults. Am J Public Health.

[27] Daugbjerg SB, Kahlmeier S, Racioppi F, Martin-Diener E, Martin B, Oja P, Bull F (2009). Promotion of physical activity in the European region: content analysis of 27 national policy documents. J Phys Act Health.

[28] National Institute for Health and Care Excellence. Walking and cycling: local measures to promote walking and cycling as forms of travel or recreation (PH41). London; 2012.

[29] 6 in 10 Adults Now Get Physical Activity by Walking. [http://www.cdc.gov/Features/VitalSigns/Walking/]. Accessed 16 October 2014.

[30] Everybody active, every day: a framework to embed physical activity into daily life. [https://www.gov.uk/government/publications/everybody-active-every-day-a-framework-to-embed-physical-activity-into-daily-life]. Accessed 5 November 2014.

[31] Hanson S, Jones A (2015). Is there evidence that walking groups have health benefits? A systematic review and meta-analysis. Br J Sports Med.

[32] Kassavou A, Turner A, French DP (2013). Do interventions to promote walking in groups increase physical activity?A meta-analysis. Int. J. Behav. Nutr. Phys. Act.

[33] Cleland CL, Tully MA, Kee F, Cupples ME (2012). The effectiveness of physical activity interventions in socio-economically disadvantaged communities: A systematic review. Prev Med.

[34] Walking for Health (2013). Walking works. Making the case to encourage greater uptake of walking as a physical activity and recognise the value and benefits of Walking for Health.

[35] Dalton A, Jones A, Ogilvie D, Petticrew M, White M, Cummins S (2013). Using spatial equity analysis in the process evaluation of environmental interventions to tackle obesity: the healthy towns programme in England. Int. J. Equity Health.

[36] A beginner’s guide to geography: Guidance and methodoloy. [http://www.ons.gov.uk/ons/guide-method/geography/beginner-s-guide/index.html]. Accessed 16 April 2014.

[37] Carr-Hill RA, Chalmers-Dixon P, Lin J, Britain G. The public health observatory handbook of health inequalities measurement. Oxford: South East Public Health Observatory; 2005.

[38] Census 2011 for England and Wales Who we are Where we live What we do. [http://www.ons.gov.uk/ons/guide-method/census/2011/index.html]. Accessed 19 September 2013.

[39] Public Health Observatories. London. [http://www.apho.org.uk/]. Accessed 19 September 2013.

[40] Active people survey. London. [http://archive.sportengland.org/research/active_people_6.aspx/active_people_6.aspx]. Accessed 27 November 2013.

[41] SPSS I (2009). PASW Statistics 18.

[42] Doyle YG, Mc Kee M, Sherriff M (2012). A model of successful ageing in British populations. Eur. J. Public Health.

[43] Pensioners living alone. Small area indicators for Joint Strategic Needs assessment. [http://www.localhealth.org.uk/#l=en;v=map9]. Accessed 16 June 2014.

[44] Farrell L, Hollingsworht B, Propper C, Shilelds MA (2013). The Socioeconomic Gradient in Physical Inactivity in England.

[45] Ogilvie D, Foster C, Rothnie H (2007). Interventions to promote walking: systematic review. BMJ.

[46] Matthews A, Brennan G, Kelly P, McAdam C, Mutrie N, Foster C (2012). Don’t wait for them to come to you, you go to them. A qualitative study of recruitment approaches in community based walking programmes in the UK. BMC Public Health.

[47] Health 2020: the European policy for health and well-being. [http://www.euro.who.int/en/health-topics/health-policy/health-2020-the-european-policy-for-health-and-well-being]. Accessed 14 April 2015.

[48] Cleland CL, Hunter RF, Tully MA, Scott D, Kee F, Donnelly M, Prior L, Cupples ME (2014). Identifying solutions to increase participation in physical activity interventions within a socio-economically disadvantaged community: A qualitative study. Int J Behav Nutr Phys Act.

[49] O’Mara-Eves A, Brunton G, McDaid G, Oliver S, Kavanagh J, Jamal F, et al. Community engagement to reduce inequalities in health: a systematic review, meta-analysis and economic analysis. Public Health Res. 2013;1(4). 25642563

[50] Unlocking the power of communities to transform lives. Community health champions: creating new relationships with patients and communities. [http://www.altogetherbetter.org.uk/home.aspx]. Accessed 20 October 2015.

